# Successful Treatment of Recurrent Pulmonary Mucormycosis in a Renal Transplant Patient: A Case Report and Literature Review

**DOI:** 10.1155/2017/1925070

**Published:** 2017-03-13

**Authors:** Morgan S. Martin, Alison A. Smith, Monica Lobo, Anil S. Paramesh

**Affiliations:** ^1^Department of Surgery, Tulane University School of Medicine, New Orleans, LA, USA; ^2^Department of Pathology, Tulane University, School of Medicine, New Orleans, LA, USA; ^3^Tulane University School of Medicine, Tulane Abdominal Transplant Institute, New Orleans, LA, USA

## Abstract

*Background.* We describe the unusual case of a recently transplanted cadaveric renal transplant recipient who presented with recurrent pulmonary mucormycosis.* Case Report. *An 18-year-old man with end stage renal disease secondary to congenital renal agenesis status after cadaveric kidney transplant 4 months before presented with acute onset of fever, hemoptysis, and back pain. The patient underwent an emergent left lower lobectomy due to the critical nature of his illness. He was also treated with amphotericin with resolution of his symptoms. One week later, he had evidence of recurrent disease on imaging with a surgical site infection. He underwent reexploration with evacuation of an empyema and debridement of a surgical site infection. He was continued on IV antifungal therapy with isavuconazonium and amphotericin. Radiographic clearance of disease with three months of treatment was apparent with no evidence of recurrence at seven-month follow-up.* Discussion.* Opportunistic infections in solid organ transplant patients represent a significant source of morbidity and mortality. Most patients are treated with prophylactic anti-infective agents. However, rare infections such as pulmonary mucormycosis remain a risk. The transplant physician must be aware of these uncommon infections and their treatment strategies, including the management of uncommon recurrent disease.

## 1. Introduction

Mucormycosis, which includes fungi in the Mucorales order such as* Rhizopus* and* Mucor, *represents a rare but aggressive infection of immunosuppressed patients. Less than one percent of solid organ transplant patients are infected; however mortality can exceed greater than 80% [1–5]. Rhinocerebral, pulmonary, gastrointestinal, and disseminated disease are the most common sites for infection [6].

Most infections occur within the first year of solid organ transplant [7]. The vast majority of patients infected by mucormycosis are male, older than age of 40 with multiple risk factors, including neutropenia, diabetes, active malignancy, and iron overloaded states. In particular, renal transplant recipients are at even high risk given the presence of comorbid diabetes, use of immunosuppressants, the frequent use of voriconazole and echinocandins to treat fungal infections in transplant patients, and uremia [3, 8]. Pulmonary mucormycosis is the most common site of infection for renal transplant recipients [9]. In addition, the southern United States has the highest incidence of mucormycosis compared to other regions of the country [5].

Mucorales have known resistance to caspofungin and voriconazole. Accordingly, amphotericin B is regarded as the first-line therapeutic agent for mucormycosis [10]. However, the use of amphotericin B usually results in renal injury and concomitant loss of the renal allograft. Use of posaconazole has been proposed in several studies; however it has not shown to be as effective as amphotericin B [11]. Thus, emerging treatment strategies have included extended spectrum azoles with specific activity against Mucorales with improved side effect profiles [12, 13].

Surgical debridement is essential for effective treatment of mucormycosis. Due to the aggressive behavior of this fungus in immunocompromised patients, extensive surgical debridement has appeared to be necessary in successful clearance of disease reported in various case series [3, 14, 15]. A review of the literature did not identify any studies on the recommended treatment of recurrent pulmonary mucormycosis.

We report the unusual case of a renal transplant recipient with refractory pulmonary mucormycosis. The objective of our study is to describe the management of recurrent lung mucormycosis in a recent cadaveric renal transplant recipient. This case report adds to the growing body of literature surrounding this phenomenon and to elucidating effective treatment algorithms.

## 2. Case Presentation

**Figure 1:**
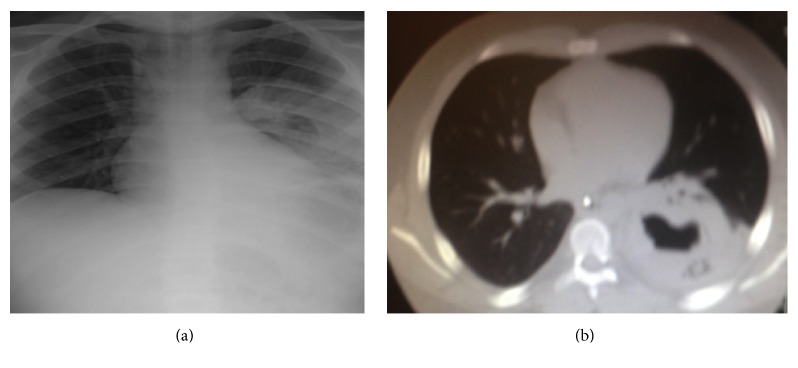
(a) Initial chest X-ray on initial presentation for an 18-year-old man status after cadaveric kidney transplant demonstrating increased opacification of the mid and lower left lung fields. (b) Computerized topography of chest/thorax for the same patient demonstrating a left lobe cavitary lesion.

An 18-year-old man with history of end stage renal disease secondary to congenital renal agenesis presented to the Emergency Department with an acute onset of hemoptysis, back pain, and fever. He had undergone a cadaveric kidney transplant four months before at a different institution. The transplant was performed without any complications, and the patient had reportedly normal kidney function. He was taking cyclosporine, mycophenolate, and prednisone for maintenance immunosuppression. He was not on any antifungal prophylaxis. The patient was not residing in a house with routine expose to soil and livestock. He denied any weight loss or recent foreign travel. His laboratory values were significant for leukocytosis with a white blood cell count of 21,900/mm^3^ and elevated creatinine. His initial chest X-ray demonstrated a consolidation of the left lower lobe (Figure 1(a)). Further imaging on chest CT showed a left lower lobe cavitary lesion (Figure 1(b)). Sputum, bronchoalveolar lavage, and blood cultures were all negative for infectious etiology. His workup was additionally negative for any additional infectious etiology. His renal biopsy showed no evidence of rejection or active infection. He was initially started on broad-spectrum antibiotics and antifungals. His immunosuppressants were held.

**Figure 2:**
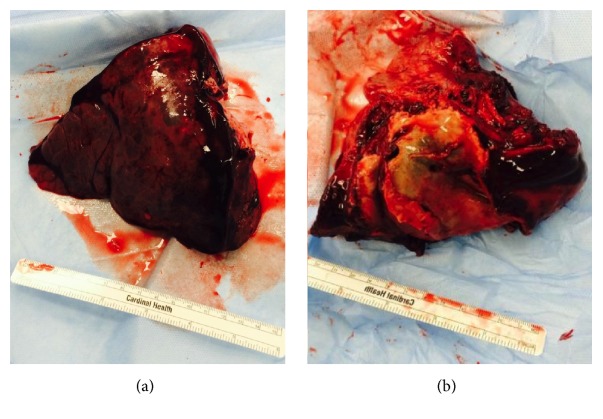
Gross pathology of consolidated fungal mass removed from lung of an 18-year-old male status after cadaveric kidney transplant. (a) Anterior and (b) posterior views of left lower lobe demonstrating a large consolidated fungal mass surrounded by area of hemorrhagic necrosis.

**Figure 3:**
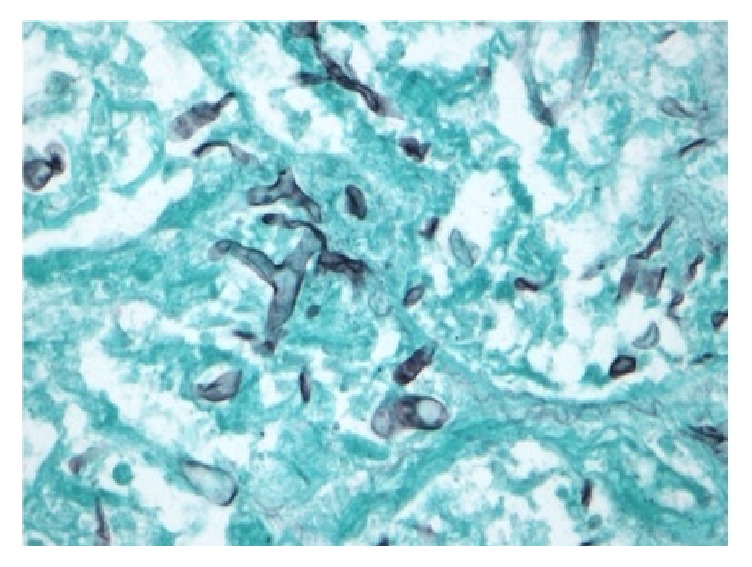
Grocott-Gomori methenamine-silver (GMS) stained tissue section of resected lung from an 18-year-old with recent kidney transplant highlighting aseptate hyphae morphologically resembling fungal class* Zygomycetes* (400x).

He was taken for an open thoracotomy with a left lower lobectomy one week after initial presentation due to the critical nature of his illness. Intraoperatively, he was found to have a solitary consolidated fungal mass surrounded by an area of hemorrhagic necrosis (Figure 2). Final pathology showed* Rhizopus* species (Figure 3). Treatment was initiated with IV liposomal amphotericin B 50 mg daily.

**Figure 4:**
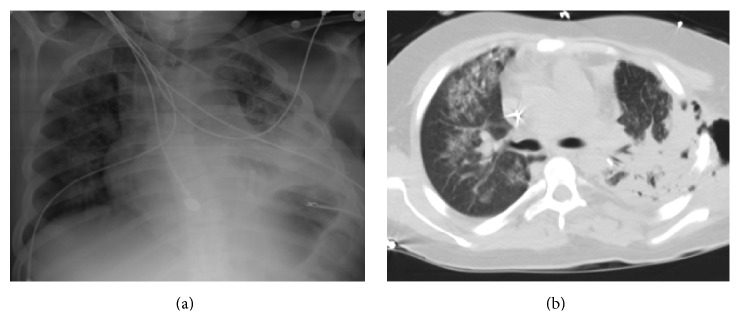
18-year-old male renal transplant recipient 24 days status after resection of left lower lobe for a fungating mass with (a) chest X-ray demonstrating left upper lobe consolidation and (b) chest computerized topography scan showing consolidation of right upper, middle, and lower lobes.

The patient demonstrated overall clinical improvement. However, one week postoperatively, the patient developed a new consolidation of his left upper lobe on chest X-ray. Repeat chest CT demonstrated an empyema. The patient also had* Rhizopus* cultured from his surgical site. He remained febrile with significant leukocytosis. He was taken to the operating room for an evacuation of an empyema and debridement of soft tissue surrounding the incision (Figures 4(a) and 4(b)).

**Figure 5:**
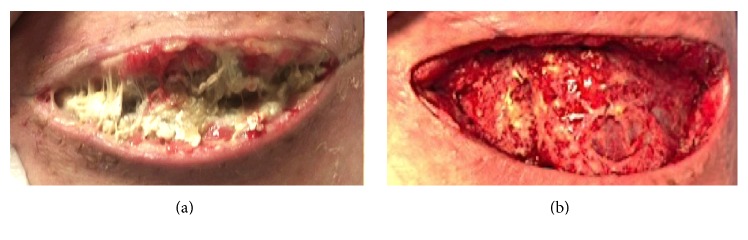
18-year-old male renal transplant recipient with pulmonary mucormycosis who developed disseminated skin infection with mucormycosis at thoracotomy site (a) and following debridement (b).

Despite aggressive surgical management of his recurrent disease, the patient continued to remain symptomatic. However, he had evidence of consolidations in both lungs (Figure 5). It was then decided to add isavuconazonium (372 mg daily). The patient improved clinically, and he was discharged home 24 days after his initial presentation.

The patient was maintained on the regimen of amphotericin B (50 mg daily) and isavuconazonium (372 mg daily) after discharge. His follow-up chest X-ray 7 months after treatment showed no evidence of residual disease. His medications were discontinued at this time. To our knowledge, he has had no further issues.

## 3. Discussion

Mucormycosis is a rare infection with a high likelihood of devastating outcomes in immunocompromised patients. Currently, there is a paucity of data on optimal treatment strategies. In particular, there are no clinical studies that demonstrate successful treatment of patients with recurrent mucormycosis. Our case report demonstrates a strategy to combat recurrent mucormycosis using salvage therapy with isavuconazonium. An initial strategy of IV liposomal amphotericin B combined with a newer medication, isavuconazonium, was employed along with aggressive surgical debridement. Despite initial efforts, the patient had evidence of recurrent pulmonary mucormycosis with dissemination of the infection to his surgical incision.

The current gold standard for mucormycosis is IV liposomal amphotericin B, surgical debridement, and, if possible, modification of risk factors [8, 10, 11]. Previous case reports have demonstrated the use of posaconazole for salvage therapy. In our study, we used isavuconazonium. Isavuconazonium is a recently developed extended spectrum azole. Studies have demonstrated clinical response to isavuconazonium with a better side effect profile than amphotericin B and posaconazole [12, 13]. In particular, the incidence of renal injury is significantly lower with isavuconazonium. Aggressive surgical debridement in combination with antifungals has shown to be a mainstay of treatment for mucormycosis. However, despite this knowledge, some case studies reviewed had high mortality rate, approaching 100% in some studies [12–17]. A summary of published reports from previous studies is presented in Table 1.

There are several important limitations to our study. First, we presented evidence from a single patient. High-quality randomized control trials are needed to further elucidate the use of isavuconazonium. Additionally, it is possible to consider that the first pneumonectomy was inadequate and contributed to the spread of the initial disease to the patient's skin.

In conclusion, we present a rare case of recurrent pulmonary mucormycosis with skin dissemination in an immunocompromised patient that was successfully managed with a combination of surgical treatment and aggressive pharmacologic therapy, including amphotericin B and isavuconazonium. It is also important to be aware of this possible diagnosis in all immunosuppressed patients and to be well-versed on potential treatment options for recurrent or refractory disease.

## Figures and Tables

**Table 1:** Summary of existing publications on mucormycosis in renal transplant recipients.

Reference	Number of patients	Organ (*N*)	Treatment	Mortality *N* (%)
Fisher et al., 1980	1	Skin (1)	AmpB, surgery	1/1 (100)
Nampoory et al., 1996	2	Lungs (1); allograft (1)	AmpB, fluconazole	—
Bakshi and Volk, 2001	1	Lungs (1)	AmpB	0/1 (0)
Godara et al., 2011	16	Rhino (9); lungs (5); allograft (1); disseminated (1)	AmpB, surgery	6/16 (37.5)
Zhao et al., 2012	1	Cutaneous (1)	AmpB, surgery	0/1 (0)
Hatahet et al., 2013	1	Disseminated (1)	AmpB, Mica, surgery	1/1 (100)
Stewart et al., 2014	1	Lungs (1)	Pos, AmpB, Mica, surgery	0/1 (0)
Patel et al., 2014	1	Lungs (1)	Pos, caspofungin	1/1 (100)
Santos et al., 2015	2	Lungs (2)	AmpB, surgery	2/2 (100)
Kursun et al., 2015	1	Rhino (1)	AmpB, surgery	—
Palejwala et al., 2016	2	Rhino (2)	AmpB, Pos, mica, Isa, surgery	2/2 100
Vaezi et al., 2016	20	Rhino (9); lungs (5); cutaneous (3); disseminated (2) pseudoaneurysm (1)	AmpB, Pos	10/20 (50)
